# Bee Bread Exhibits Higher Antimicrobial Potential Compared to Bee Pollen

**DOI:** 10.3390/antibiotics10020125

**Published:** 2021-01-28

**Authors:** Karolina Pełka, Olga Otłowska, Randy W. Worobo, Piotr Szweda

**Affiliations:** 1 Department of Pharmaceutical Technology and Biochemistry, Faculty of Chemistry, Gdańsk University of Technology, ul. G. Narutowicza 11/12, 80-233 Gdańsk, Poland; karolina.pelka@pg.edu.pl (K.P.); olga.otlowska@pg.edu.pl (O.O.); 2 Department of Food Science, Cornell University, Ithaca, NY 14853, USA; rww8@cornell.edu

**Keywords:** bee pollen, bee bread, *Staphylococcus aureus*, antimicrobial activity

## Abstract

This study aimed at investigation of the antimicrobial potential of ethanolic extracts of bee bread (BB) and bee pollen (BP) and suspensions of these products in MHB (Mueller Hinton Broth). We covered 30 samples of BP and 19 samples of BB harvested in Polish apiaries. Slightly lower activity was observed against Gram-negative bacteria compared to Gram-positive staphylococci. BB extracts exhibited higher inhibitory potential with minimum inhibitory concentration (MIC) values in the range from 2.5 to 10% (*v*/*v*) against *Staphylococcus aureus* ATCC 25923 and ATCC 29213. Most active BB extracts, namely, BB6, BB11 and BB19, effectively inhibited growth of clinical isolates of *S. aureus* (*n* = 9), including MRSA (*methicillin resistant Staphylococcus aureus*) strains (*n* = 3) at concentrations ranging from 2.5 to 5.0% (*v*/*v*). Minimal bactericidal concentration (MBC) values were in the same range of concentrations; however, a shift from 2.5 to 5.0% (*v*/*v*) was observed for some products. The most active BP extracts inhibited the growth of reference strains of *S. aureus* at a concentration of 5% (*v*/*v*). Up to the concentration of 20% (*v*/*v*) three and seven BP extracts were not able to inhibit the growth of *S. aureus* ATCC 29213 and *S. aureus* ATCC 25923 respectively. The growth of staphylococci was also importantly inhibited in suspensions of the products in MHB. No correlation between phenolic content and antimicrobial activity was observed.

## 1. Introduction

A healthy microbiota is absolutely crucial for a developing bee colony. The whole surface of the hive, including the combs, is covered with propolis—a highly agglutinative, resinous substance of complex chemical composition that is collected by bees from flower and leaf buds. Some of its ingredients, mainly polyphenols and flavonoids, exhibit high antimicrobial activity and protect the bee colony against dangerous pathogens from the hive environment [1,2,3,4]. A high concentration of sugars (high osmotic pressure), bee defensins, enzymatic production of hydrogen peroxide and phytochemicals protect the honey against microbial spoilage and development of pathogenic microorganisms for bees [5,6,7,8,9,10]. Bees also collect pollen, which is a primary source of protein and fat for larvae and young bees [11]. Pollen is the male reproductive cell of the flower. Beekeepers collect pollen granules and sell it on the market. Recently, this product has gained popularity among consumers. Bee pollen (BP) is considered as a healthy/functional food; it contains all the essential amino acids needed by bees, but also human bodies. It is a rich source of fatty acids, vitamins and microelements [12,13]. Moreover, it exhibits a wide range of therapeutic properties, such us antimicrobial, antioxidant, anti-radiation, anti-inflammatory, anti-tumor, hepatoprotective and chemopreventive/chemoprotective benefits [12,13,14,15,16]. High contents of polyphenols and other ingredients that exhibit antibacterial and antifungal activity only partly inhibit the growth of microorganisms and protect pollen grains against microbial spoilage. The drying process is required to ensure the microbiological safety of BP that is proposed for consumers. It is important to perform this process in mild conditions, at ambient temperature to protect the health beneficial ingredients of this product.

As mentioned above, the BP collected by bees is susceptible to microbiological deterioration. In fact, for bees the pollen is only the raw material for production of bee bread (BB). In hives, some of the BB is stored in the wells of the honeycomb through the winter and in the spring it is used as food for new populations of bee larvae. Bee workers collect pollen from plant anthers, mix it with a small dose of the secretion from salivary glands and/or nectar and place it in specific baskets (corbiculae) which are situated on the tibia of their hind legs. These pollen loads are transported to the hive. Subsequently, pollen loads are packed in the honeycomb cells, and covered with a thin layer of honey and a waxy lid. In these anaerobic conditions, bee pollen undergoes fermentation and biochemical changes that also constitute a method of preservation for the final product of the process—bee bread [16,17,18,19]. The exact mechanism of the biotransformation processes of BP to BB remains not fully understood. However, it is known that different enzymes from bees’ glands (e.g., amylases that are responsible for starch hydrolysis) and bacteria that are present in bees’ saliva and on the surfaces of pollen loads (mostly lactic acid bacteria—LAB but also bacteria of the *Pseudomonas* genus and yeast of the *Saccharomyces* genus) are crucial for this process [16,18,20,21]. The development of the population of LAB, hydrolysis of triacylglycerols and production of lactic acid and probably other metabolites of antimicrobial activity (e.g., bacteriocins) is certainly of primary importance for preservation of BB [20,21]. Additions of honey and polyphenols that are present in the raw material (BP) enhance the antimicrobial potential of BB and allow for long term storage of this product in the hive.

A healthy microbiota is crucial for bee larvae and young bees that are fed with BB. Another important and interesting aspect is the ability of using the antimicrobial potential of BB and BP for prophylaxis and treatment of bacterial and fungal infections of humans and animals. The primary goals of this study were the assessment and comparison of the antimicrobial abilities of ethanolic extracts of ingredients of BP and BB produced in Polish apiaries.

## 2. Results

The outcomes of this study revealed differences in the antimicrobial activity of ethanolic extracts from BB and BP. Moreover, slight differences in the susceptibility of Gram-positive and Gram-negative bacteria were also observed (Table 1). Gram-negative *P. aeruginosa*, and particularly *Escherichia coli*, exhibited higher levels of resistance compared to Gram-positive staphylococci. In the case of both *Staphylococcus aureus* reference strains, minimum inhibitory concentration (MIC) values of BB extracts were in the range of concentrations from 2.5 to 10% (*v*/*v*), and from 5 to 10% (*v*/*v*) for *Staphylococcus epidermidis* ATCC 12228. The highest activity (MIC = 2.5% (*v*/*v*)) was observed for three extracts (against *S. aureus* ATCC 25923) and for 12 products (against *S. aureus* ATCC 29213). At least 10% and 20% (*v*/*v*) concentrations were required for growth inhibition of *P. aeruginosa* ATCC 27853 and *E. coli* ATCC 25922, respectively. The highest susceptibility to the activity of extracts produced from BP was observed with *S. epidermidis* ATCC 12228; three out of thirty tested extracts (assigned with numbers 11, 15 and 17) inhibited the growth of this strain at a concentration of 5% (*v*/*v*); MIC values for other extracts were 10 or 20% (*v*/*v*). Two products, with numbers 15 and 20, effectively inhibited the growth of *S. aureus* ATCC 29213 at concentrations equal to 5% (*v*/*v*). However, six BP extracts did not exhibit any activity against this strain up to the concentration of 20% (*v*/*v*). At least 10% (*v*/*v*) concentration of BP extracts was necessary for growth inhibition of *S. aureus* ATCC 25923, and one of these extracts was not active even at the highest investigated concentration of 20% (*v*/*v*). The effectiveness of BP extracts against *P. aeruginosa* ATCC 27853 was comparable to the activity of extracts produced from BB, except one (not active up to the concentration of 20% (*v*/*v*)); these products inhibited the growth of this strain at concentrations equal to 10 or 20% (*v*/*v*). *E. coli* ATCC 25922 exhibited lower susceptibility to BP extracts; 14 products did not exhibit activity up to the concentration of 20% (*v*/*v*); and the MIC for other products (*n* = 16) was 20% (*v*/*v*). As is shown in Table 1, for many of the extracts, particularly produced from BP, a shift of minimal bactericidal concentration (MBC) values compared to MIC was observed—higher concentrations were necessary to achieve a bactericidal effect compared to growth inhibition. Clear differences were observed for *S. aureus* ATCC 25923; MBC values of 23 products were higher compared to MIC. Twenty BP extracts did not exhibit bactericidal activity against *S. epidermidis* ATCC 12228 up to the concentration of 20% (*v*/*v*) and the same values of MBC and MIC parameters were found for only three extracts. Only four BP extracts effectively killed *E. coli* ATCC 25922 at the highest investigated concentration of 20% (*v*/*v*). Considering two other reference strains of bacteria, the shift of MBC values compared to MIC was observed for 9 and 12 BP extracts for *P. aeruginosa* ATCC 27853 and *S. aureus* ATCC 29213, respectively. The differences in MBC and MIC values for BB extracts were also observed. However, the differences were not so evident. The MBC values for all staphylococci and *P. aeruginosa* ATCC 27853 were ≤20% (*v*/*v*) and only six BB extracts were not capable of killing *E. coli* ATCC 25922 cells at the highest investigated concentration of 20% (*v*/*v*). Similarly, in the case of BP extracts, the most important differences in MIC and MBC values were found for *S. epidermidis* ATCC 12228—15 out of 19 of the extracts tested. On the other hand, only four extracts exhibited differences in effective inhibitory and bactericidal activity against *P. aeruginosa* ATCC 27853. Considering *S. aureus* reference strains differences between MBC and MIC values were noted for 10 and 8 BB extracts for *S. aureus* ATCC 29213 and *S. aureus* ATCC 25923, respectively.

The high anti-staphylococcal potential of extracts produced from BB and BP collected in Polish apiaries was confirmed for clinical isolates, including six methicillin-susceptible strains (MSSA) and three isolates that were methicillin-resistant (MRSA) (Table 2). The investigation was performed for three BB extracts with numbers: 4, 11 and 14 and three extracts produced from pollen, with numbers 9, 15 and 20. These extracts were found to be highly active against reference strains of staphylococci. The results of the assay confirmed the high anti-staphylococcal potential of the extract produced from both types of raw materials, namely, BP and BB. However, similarly to the case of reference strains of staphylococci, BB extracts exhibited higher inhibitory potential with MIC values in the range from 2.5 to 5.0% (*v*/*v*) against all strains tested, including MRSA isolates. In addition, the MBC values were in the same range of concentrations. However, in some cases, a two times higher concentration of BB extract was necessary to achieve a bactericidal effect in comparison to MIC value (shift from 2.5 to 5% (*v*/*v*)). The MIC values of BP extracts against clinical isolates of *S. aureus* were in the range from 5 to 10% (*v*/*v*); the products with numbers 15 and 20 generally exhibited higher activity compared to BP9. The MBC values were in the range from 5 to 20% (*v*/*v*). However, in most cases the values of MIC and MBC were exactly the same. For BP9, BP15 and BP20 extracts, the shift of MBC value compared to MIC was observed for only two, one and two strains, respectively.

Using a slightly modified Folin–Ciocalteu method [22], we determined total phenolic content in the produced extracts (Table 1). The content of polyphenols, expressed as milligrams of gallic acid equivalent (GAE) per gram of the product, ranged from 13.94 to 21.054 mg GAE/g for BP extracts and from 16.877 to 20.179 mg GAE/g for BB extracts. Most importantly, no correlation between the antibacterial efficacy and concentrations of polyphenols was observed. In the study of Markiewicz-Żukowska [23], who investigated three samples of BB collected by Polish beekeepers, the total phenolic content (TPC) values ranged from 32.78 to 37.15 mg GAE/g. These differences in concentration for polyphenols can be explained by some differences in extractions procedures that were applied in both studies.

**Kinetics of the bactericidal action of BP and BB extracts.**

**Figure 1:**
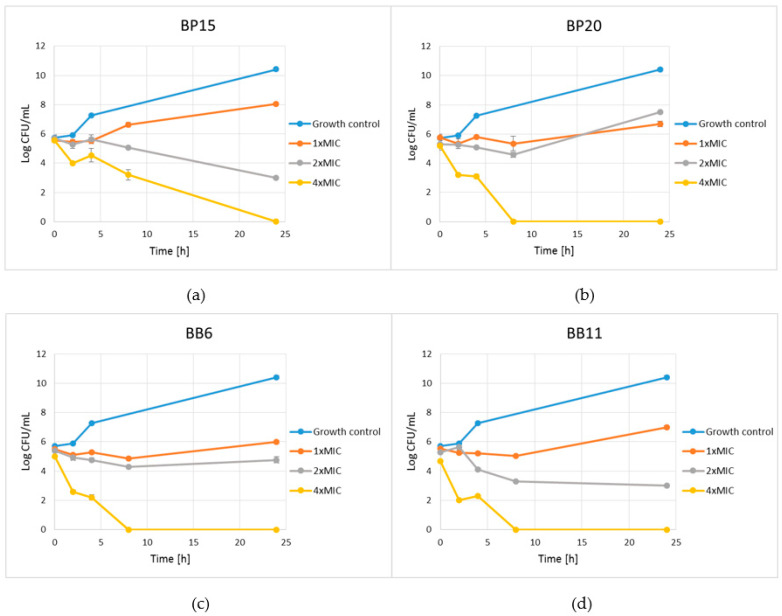
Kill-time assay for selected ethanolic extracts of bee pollen and bee bread tested against *S. aureus* ATCC 29213 at or above the MIC. The growth control contained no extracts; (**a**) BP15, (**b**) BP20, (**c**) BB6, (**d**) BB11. The results are presented as means ± SDs (*n* = 3). Data without error bars indicate that the SDs are too small to be observed on the graph.

Four selected extracts, two produced from BP (assigned as 15 and 20) and two produced from BB (assigned with numbers 6 and 11), were used for the determination of the bactericidal effects of ingredients extracted from the raw materials with ethanol (70% *v*/*v*) against *S. aureus* ATCC 25923. As expected, at MICs, all products resulted in only a growth inhibition effect. Slightly higher—though still classified as inhibitory—activity was observed for three products, namely, BB6, BB11 and BP15, when used at concentrations equal to 2 × MIC; extract from bee pollen number 15 exhibited lower antibacterial activity. At concertation 4 × MIC, the extracts from both bee bread samples, and surprisingly BP15, resulted in completely bactericidal effects after 8 h of incubation. Activity of the extract BP20 was considerably lower. However, complete elimination of living cells of bacteria was achieved after 24 h of incubation (Figure 1).

**Figure 2:**
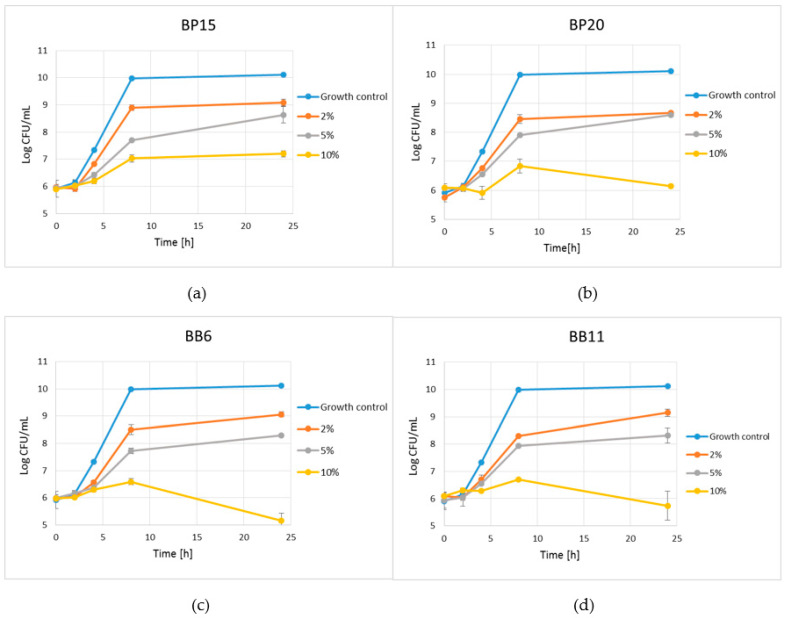
Kill-time assay for suspensions of selected products tested against *S. aureus* ATCC 29213 in final concentrations of 2%, 5% and 10% (*v*/*w*). The growth control did not contain any product; (**a**) BP15, (**b**) BP20, (**c**) BB6, (**d**) BB11. The results are presented as means ± SDs (*n* = 3). Data without error bars indicate that the SDs are too small to be observed on the graph.

A promising antimicrobial effect was also observed during incubation of *S. aureus* ATCC 29213 cells in suspensions of selected products in MHB medium (Figure 2). At a concentration of 2.0% (*w*/*v*), after 24 h of incubation, all products inhibited the growth of the bacterial cells by about 90%, compared to the control sample. In all cases, only a slightly higher growth inhibition effect was observed for suspensions containing 5.0% of the products. Much better effectiveness was achieved in suspensions containing 10% (*w*/*v*) of the products. After 24 h of incubation, the decreased level of growth inhibition of more than four log cycles was observed for both bee bread samples, from LogCFU/mL 10.1—control sample to 5.7 and 5.2 for BB11 and BB6, respectively. An important growth inhibition level, from LogCFU/mL 10.1—control sample to 6.1—exhibited 10% (*w*/*v*) suspension of BP20. The efficiency of BP15 was slightly lower. However, even in the case of this product, about 99.9% (about three log cycles, from 10.1 to 7.2.) growth inhibition was observed after 24 h of incubation.

## 3. Discussion

Antimicrobial potential of bee honey and propolis has been known since ancient times. These two products belonged to the most important and most common antimicrobial agents of folk medicine that were used for treating of infections, particularly for treatment of difficult to heal infected wounds [1,7,24]. The antimicrobial potential of bee pollen and bee bread is definitely less known and less investigated [16]. We still do know if there are important differences in antibacterial potential of BB and BP, spectrum of activity also remains not clear, some authors suggest that Gram-positive bacteria exhibit higher susceptibility and, which is most important, ingredients that are crucial for antimicrobial activity of these product are not identified. To date, most research has focused on the high nutritional value of these products. Due to high content of health beneficial ingredients, including vitamins [25,26,27], micro and macro-elements [12,28] fatty acids [23,29,30,31], amino acids [32,33] and also different groups of phytochemicals—mostly important as antioxidants [16,23,34], BP and BB are considered functional foods. However, the outcomes of recent research showed promising antimicrobial potential of BB and BP produced in apiaries located in different regions of the world. In this study, we have investigated relatively large numbers of samples of both products, namely, 30 samples of BP and 19 samples of BB. To our best knowledge presented to date, reports were based on analysis of lower numbers of samples, in some cases singular products. Analyses of properties of a large group of products led us to conclusion that BB extracts (MIC against staphylococci in the range of concentrations from 2.5 to 10% *v*/*v*) exhibit higher antimicrobial potential compared to the activity of BP extracts (MIC values against staphylococci in the range of concentrations from 5 to >20% *v*/*v*). We assume that it is a consequence of the process of fermentation that is the base for transformation of raw material—BP to the final product—BB. During the fermentation process the enzymes that were added to the raw material by bees and also produced by bacteria that were present on the surface of pollen and in the bees’ saliva result in partial digestion of biopolymers (mostly polysaccharides) that cover pollen grains. As a consequence, the ingredients of pollen located inside the grains are more accessible to the solvents and easier to extract. This hypothesis seems to be not supported by the results of investigation of TPC—the extracts produced from both products exhibit comparable values of this parameter. However, Markiewicz-Żukowska and coworkers [23] revealed that BB extracts contain many other components that exhibit antimicrobial activity except for polyphenols. The most important of them seem to be aliphatic acids. Aliphatic acids were found to be the predominant components of the extracts investigated by the group of Markiewicz-Żukowska (62.32 ± 7.0%) and unsaturated, α-linolenic, linoleic, oleic and 11,14,17-eicosatrienoic acids formed more than a half of them (40.63 ± 4.5%). Moreover, Vasquez and Olofsson [21] and Iorizzo [35,36] revealed the presence in BB and BP the presence of Lactic Acid Bacteria (LAB) e.g., *Lactobacillus kunkeei* [35], *L. plantarum* [36] that are able to produce metabolites (e.g., bacteriocins), which exhibit high antimicrobial potential. These bacteria readily grow within the first step of biotransformation of BP to BB—for approximately two weeks [21]. Within this time, they produce and transport to the maturing BB antimicrobial metabolites that include lactic acid and bacteriocins, but also participate in lipid hydrolysis and production of aliphatic acids. All these aspects together are likely the reason for higher antimicrobial activity of BB compared to BP. BB is absolutely necessary for feeding young bee larvae in early spring. Thus high antimicrobial potential (higher than bee pollen) is important benefit of this product, which in fact allows it to be stored in hive during the winter.

In agreement with results presented herein, most of the results presented by other authors confirm higher susceptibility of Gram-positive bacteria to the components of BP or BB extracts. Important higher susceptibility of *S. aureus* compared to *P. aeruginosa* and/or *E.coli* against BP extracts presented for example: Velasquez and coworkers [35] who investigated extract produced from sixteen samples of Chilean pollen samples [37], Pascola and coworkers who investigated eight products from Portugal and Spain [38], Karadal et al. (5 BP samples from Turkey) [39], Abouda et al., (four pollens collected in Morocco) [40], Khider (three Egyptian BP samples) [41] and Graikou who analyzed biological properties of one Greek BP [42]. The same differences in activity against staphylococci and Gram-negative bacteria have been shown for extracts produced form Romanian [43], Malaysian [44], and some of Moroccan samples of BB [40]. However, it should be clearly noticed that currently it is too early to propose a general rule concerning BP or BB extracts against Gram-positive or Gram-negative bacteria. As it is presented above, the number of currently available data is very limited. Moreover, some authors observed contradictory results, e.g., the group of Ivanisova who investigated antibacterial potential of Ukrainian BB [45]. The outcomes of some investigations suggest that method of extraction, such as solvent, can importantly affect different aspects of biological properties of produced extracts, including antimicrobial activity [46,47,48].

An important advantage of extracts produced from samples of Polish BB and also BP is high efficacy against clinical isolates of *S. aureus*, including MRSA strains. This part of the study additionally confirmed a bit higher inhibitory and also bactericidal activity of BB extracts (MIC and MBC values ranged from 2.5 to 5.0% (*v*/*v*)) compared to extracts produced from BP (MIC and MBC values ranged from 5.0 to 10.0% (*v*/*v*)). In our previous study we revealed high activity of honey and propolis produced in Polish apiaries against *S. aureus* isolates that exhibit MRSA phenotype [3,8]. All these results support the idea of application of bee products as alternative antibacterial agents, including treatment of infections caused by resistant strains. Of course, we realize that in clinical scenario potential application of bee products, similarly as in the case of many other natural products such as essential oils or plant extracts, is limited to topical infections (e.g., treatment of infected wounds or skin infections).

Some important conclusions come also from the analysis of the bactericidal potential of the extracts that was performed with the time-kill kinetic assays. Firstly, it has been shown that achievement of bactericidal effect requires using four times higher concentration compared to the MIC. In microdilution assay the MBC values for most products were two times higher compared to MIC. In our opinion, some differences in the conditions of these two assays should explain the observed differences—shaking (intense aeration) in time-kill kinetic assays is likely the main reason for higher resistance of the staphylococci to the activity of ingredients of the extracts. However, the observed results generally confirm high anti-staphylococcal potential of BP and particularly BB extracts and additionally support the need for more advanced studies focusing on the application of these products for treatment of bacterial infections. Moreover, Olczyk and coworkers revealed that bee pollen ointment may affect the wound healing process of burn wounds, preventing infection of the newly formed tissue [49].

As it was mentioned above we did not observe any correlation between the TPC and antimicrobial activity of produced extracts. However, the values of TPC were quite similar to the results presented by other authors who investigated BP or BB from other different geographical locations e.g., Poland [23], Portugal [50,51], Greece [42], Romania and India [52], or Chile [53].

The last, but not least aspect of our study was to check if growth of staphylococci is affected in water suspension of selected samples of BP and BB (the samples of the products that were used for production of most active extracts were used in this part of the study). In all cases the suspensions containing only 2.5% *w*/*v* of the product efficiently inhibited the growth *S. aureus* ATCC 29213 cells compared to the control. Except for BP20, increase of product concentration resulted in higher growth inhibition activity in concentration dependent manner. At concentration of 10% (*w*/*v*) all products inhibited the growth of bacteria in at least 3 log cycles compared the control. Again, a bit higher activity was observed for BB samples. This part of research clearly indicates that both bee bread and bee pollen contain some antimicrobial components, including polyphenols, fatty acids and bacterial metabolites (produced by endogenous microflora of these raw materials), which are crucial for bee health and also for abilities of storage of some amounts of the BB in the hives during the winter period. It would be very interesting to use this potential to obtain products (extracts) that could be used as antibacterial therapeutic agents.

## 4. Materials and Methods

### 4.1. Bee Pollen (BP) and Bee Bread (BB) Samples

The study covered 30 samples of BP and 19 samples of BB. The products were harvested between 1 May and 15 September of 2019 in apiaries located in different regions of Poland (Table 1). The pollen loads were collected in special pollen traps that were installed in front of the hive entrance. All samples of BP, even the products that were delivered directly by beekeepers were dried (it protected the product against microbial spoil). The BB was recovered directly from honeycombs in late summer or autumn 2019; thus, only mature bee bread was used for the study. All products were not older than eight months counting form the date of harvesting to the date of preparing the extracts or using them for other assays presented herein. In the case of some products that were bought in shops, we were not able to establish geographic origins—they were not declared by the sellers. All products were stored in the dark, BP was kept at ambient temperature and BB was stored at 4 °C.

### 4.2. Chemicals and Reagents

All chemicals and reagents were purchased from commercial sources. The Folin–Ciocalteu reagent PBS, methanol, gallic acid and sodium carbonate were purchased from Merck (Darmstadt, Germany) and ethanol was bought from (POCH, Gliwice, Poland). TheMilli-Q Advantage A10 system (Millipore, Billerica, MA, USA) was used for production of ultrapure H_2_O (18.0 MΩ) and Genesys 20 spectrophotometer (Thermo Scientific, Waltham, MA, USA) was used for measurement of absorbance in Folin–Ciocalteu assay.

### 4.3. Bacterial Strains and Media

Five reference strains of bacteria, *S. aureus* ATCC 25923, *S. aureus* ATCC 29213, *S. epidermidis* ATCC 12228, *P. aeruginosa* ATCC 27853 and *E. coli* ATCC 25922, were applied for preliminary assessments of the antimicrobial potential of all produced BP and BB ethanolic extracts Subsequently anti-staphylococcal activity of selected extracts was evaluated against 6 MSSA (*methicillin-susceptible Staphylococcus aureus*) and 3 MRSA *(methicillin-resistant Staphylococcus aureus*) isolates from patients of the hospital of Medical University of Gdańsk, that suffered with different infections (Table 3). Bacteria were routinely grown on Luria-Bertani Agar (LA, Sigma Aldrich, Schnelldorf, Germany). The determination of the values of Minimum Inhibitory Concentration (MIC) was performed in Mueller-Hinton Broth (MHB, Sigma Aldrich) and for determination of Minimum Bactericidal Concentrations (MBC) the cells were cultivated on the selective Baird Parker Agar plates (Biomaxima, Lublin, Poland). The reference strain *S. aureus* ATCC 29213 was used for antimicrobial potential evaluation of both extracts and suspensions of selected products in kinetic time-kill assay.

### 4.4. Preparation of BP and BB Ethanolic Extracts

The rotary platform shaker was used for efficient extraction of active components from raw materials (BP or BB). The suspensions of BP/BB in 70% ethanol at *v*/*w* ratio 7:1 were shaken (100 rpm) for 2 h at ambient temperature. Next, the suspensions were centrifuged (2290× *g*, 20 min) and the obtained supernatants were filtered through the sterile, 0.22 μm pore-sized filters (obtained from Millipore, Burlington, MA, USA). Finally, clear (not cloudy) and sterile extracts were obtained and used in subsequent studies.

### 4.5. Investigation of Antimicrobial Potential of Alcoholic Extracts of BP and BB—Determination of Values of MIC (Minimum Inhibitory Concentration) and MBC (Minimum Bactericidal Concentration)

The minimum inhibitory concentrations (MICs) were determined by the two-fold broth microdilution method according to the CLSI standard methodology [54]. All bacterial strains used for the assay (both reference strains and clinical isolates) were cultivated overnight at 37 °C on Luria-Bertani Agar plates. The bacterial suspension of two to three colonies (taken from the L-B Agar plates) in PBS buffer (pH = 7.4) was adjusted to the optical density of OD_600_ = 0.1 and diluted in MHB medium at a ratio of 1:100 *v*/*v* to the final cell concentration of approximately 1.0 × 10^6^ CFU/mL.

Serial two-fold dilutions of the tested extracts of BP or BB (in the range of concentrations from 0.078–40% (*v*/*v*)) were prepared in 96-well microtitration plates in the final volume of 100 μL of MHB medium (CMHB2). In the next step of the assay, the solutions of the BP and BB extracts in the wells were inoculated with an equal volume (100 µL) of suspension of bacterial cells (prepared as presented above). The final concentrations of inoculated extracts ranged from 0.039% to 20.0% (*v*/*v*). Ten different concentrations of the extracts were tested: 20.0, 10.0, 5.0, 2.5, 1.25, 0.625, 0.315, 0.156, 0.078 and 0.039% (*v*/*v*) in the columns 1 to 10 of the microtitration plates. Column 11 contained 200 µL of inoculum (growth control in the medium free of antibacterial agents), and column 12 contained 200 µL of the MHB broth only (as control of sterility of the medium). The plates were incubated 24 h under static conditions at 37 °C. As color and solubility of BP and BB extracts interfered with growth measurement it was necessary to perform the resazurin test. After incubation, resazurin solution (0.015% in PBS buffer) was added to all wells (30 µL), and plates were incubated at 37 °C in the dark for the next two hours. The lowest concentration of the extract with no color change (blue resazurin color remained unchanged) was taken as a MIC value. The same method was applied for determination of activity of the solvent (70% ethanol) that was used for preparing of the extracts and no inhibitory activity was observed even in the wells of column 1, where the concentration of the EtOH was 14%. A sterile 48-well microtiter plate replicator was used for transferring a small volume of each dilution used for MIC assay on Baird-Parker agar plates. Subsequently the plates were incubated for 24 h at 37 °C and growth of characteristic black colonies of *S. aureus* was analyzed. The lowest concentrations of the extracts, where no growth of the colonies was observed, were assigned as MBC.

### 4.6. Time-Kill Assay—Determination of Kinetic of Bactericidal Effects of BP and BB Extracts and Suspensions of Raw Materials against Staphylococci

The kinetic time-kill assay was performed for two most active extracts of BP and BB and also for suspensions of raw materials (two samples of each BP and BB) used for preparing of these extracts. The selected extracts were added to the suspensions of *S. aureus* ATCC 29213 (approx. cell density 1.5 × 10^6^ CFU/mL) in MHB broth to the final concentration equal to MIC, 2 × MIC and 4 × MIC. In the case of determination of activity of raw materials, the suspension of *S. aureus* ATCC 29213 was supplemented with bee pollen or bee bread to the final concertation of 2.0, 5.0 or 10.0% *w*/*v*. The treated suspensions of *S. aureus* ATCC 29213 were incubated at 37 °C with shaking. The number of the cells of bacteria that survived treatment for 0, 2, 4, 8 and 24 h was determined by plating 10-fold dilutions of the suspensions on Baird-Parker agar plates and incubating the plates at 37 °C for 24 h. The number of the cells in the control suspension, without extract/product addition, was also determined as a control of growth kinetic of *S. aureus* ATCC 29213.

### 4.7. Total Phenolics Determination

The slightly modified Folin–Ciocalteu method [22] was used for determination of total content of phenolic compounds in produced ethanolic extracts of BP and BB. Briefly, 50 µL of Folin–Ciocalteu reagent diluted 1:10 with de-ionized ultrapure water was mixed with 10 μL of the extract. After 5 min of incubation, 40 µL of Na_2_CO_3_ solution (7.5%) was added to the mixture. Following shaking 100 µL of ultrapure water was added (to the final volume of 200 µL) and the mixture was incubated for 30 min at ambient temperature. The color intensity—absorbance at 725 nm—was measured using microplate reader (Synergy™ HT BioTek Instruments, Winooski, VT, USA). The calibration curve was prepared with fresh gallic acid standard solutions in the range of concentrations from 1.56 to 50.00 µg GAE/mL. The content of phenolic compounds in BP and BB extracts was expressed as milligrams of gallic acid equivalent (GAE) per gram of the product. All measurements were performed in triplicate.

## 5. Conclusions

The outcomes of the study revealed high antimicrobial potential for ethanolic (70% *v*/*v*) extracts of BP and BB produced in Polish apiaries. Moreover, we observed high growth inhibitory activity of suspensions of BB and BP against *S. aureus*. In both cases (extracts and raw products—suspensions), BB exhibited importantly higher activity and Gram-positive bacteria exhibited higher susceptibility. The extracts exhibited high activity against clinical isolates of *S. aureus*, including MRSA strains, which supports the need for further investigation of possibilities of the applications of BP and BB and products based on these raw materials (extracts, ointments, etc.) as antimicrobial agents.

## Figures and Tables

**Table 1:** Antimicrobial activity and total phenolic content (TPC) of extracts produced from bee pollen (BP) and bee bread (BB) samples harvested in Polish apiaries.

Product	*S. aureus* ATCC 253923		*S. aureus* ATCC 29213		*S. epidermidis* ATCC 12228		*E. coli* ATCC 25922		*P. aeruginosa* ATCC 27853		TPC [mg GAE/g]	Sample Location
	MIC (*v*/*w*) [%]	MBC (*v*/*w*) [%]	MIC (*v*/*w*) [%]	MBC (*v*/*w*) [%]	MIC (*v*/*w*) [%]	MBC (*v*/*w*) [%]	MIC (*v*/*w*) [%]	MBC (*v*/*w*) [%]	MIC (*v*/*w*) [%]	MBC (*v*/*w*) [%]		
BP1	10	>20	10	20	10	>20	>20	>20	20	20	21.05 ± 0.09	Szczytno ^a^
BP2	20	>20	20	>20	20	>20	>20	>20	10	20	20.61 ± 0.16	Mielec ^a^
BP3	10	20	>20	>20	10	>20	>20	>20	10	20	20.92 ± 0.18	Mielec ^a^
BP4	20	>20	>20	>20	20	>20	20	>20	20	20	20.96 ± 0.23	Mielec ^a^
BP5	10	>20	10	10	10	>20	20	20	20	20	20.74 ± 0.06	Gdańsk ^a^
BP6	10	20	20	20	20	>20	20	>20	20	20	20.37 ± 0.17	Kozaki
BP7	10	20	10	20	10	20	20	20	20	20	20.81 ± 0.09	Koryciny
BP8	20	>20	>20	>20	20	>20	20	>20	>20	>20	20.76 ± 0.28	Stróże
BP9	10	20	10	10	10	10	20	>20	20	20	20.49 ± 0.43	Czarne
BP10	10	20	20	20	10	>20	20	>20	20	20	20.99 ± 0.16	Brusy
BP11	10	20	10	20	5	20	20	>20	10	20	20.40 ± 0.06	Bielsko-Biała ^a^
BP12	20	>20	>20	>20	20	>20	>20	>20	10	20	19.95 ± 0.22	Gdańsk ^a^
BP13	10	20	10	20	10	20	>20	>20	10	10	20.93 ± 0.19	Cychry
BP14	10	>20	20	>20	20	20	>20	>20	10	20	19.91 ± 0.18	Malbork ^a^
BP15	10	10	5	5	5	10	20	20	10	10	19.32 ± 0.12	Niżna Łąka
BP16	20	20	10	20	10	10	>20	>20	20	20	20.39 ± 0.35	Siedlce ^a^
BP17	20	20	20	20	5	20	20	>20	10	20	16.38 ± 0.35	Miłogoszcz
BP18	20	>20	>20	>20	10	20	20	>20	10	20	13.95 ± 0.50	Miłogoszcz
BP19	20	>20	10	20	20	>20	>20	>20	10	20	19.81 ± 0.35	Miłogoszcz
BP20	10	10	5	5	10	20	>20	>20	10	10	19.60 ± 0.41	Modzele
BP21	20	>20	>20	>20	20	>20	>20	>20	20	20	16.17 ± 0.81	Miłogoszcz
BP22	20	20	20	>20	10	>20	20	>20	10	10	20.56 ± 0.34	Wałcz ^a^
BP23	20	20	10	10	10	>20	20	>20	20	20	14.79 ± 0.20	Miłogoszcz
BP24	10	20	10	20	10	>20	20	20	10	10	20.24 ± 0.18	Czaplinek
BP25	10	20	10	20	20	>20	20	>20	10	10	20.95 ± 0.32	Stanisławowo
BP26	10	>20	10	10	20	>20	20	>20	20	20	19.08 ± 0.14	Mielec ^a^
BP27	>20	>20	20	>20	20	>20	>20	>20	20	20	20.40 ± 0.08	Miłogoszcz
BP28	20	>20	20	>20	20	>20	>20	>20	20	20	18.34 ± 0.25	Mielec ^a^
BP29	10	20	10	10	10	>20	>20	>20	10	20	16.64 ± 0.21	Miłogoszcz
BP30	10	20	10	10	20	>20	>20	>20	10	10	18.94 ± 0.21	Pelplin ^a^
BB1	10	10	2.5	5	5	10	20	>20	10	20	20.18 ± 1.22	Legnica ^a^
BB2	5	5	2.5	5	5	20	20	20	10	10	19.47 ± 0.38	Malbork ^a^
BB3	10	20	5	5	5	10	20	20	10	10	16.88 ± 0.52	Bielsko-Biała ^a^
BB4	5	5	2.5	5	5	10	20	>20	10	10	19.42 ± 0.31	Cychry
BB5	5	5	2.5	5	5	10	20	20	10	10	17.65 ± 0.29	Stanisławowo
BB6	2.5	5	2.5	5	5	10	20	>20	10	10	17.03 ± 0.41	Czaplinek
BB7	5	10	2.5	2.5	5	10	20	20	10	20	18.89 ± 0.43	Mielec ^a^
BB8	5	5	5	5	5	10	20	>20	10	20	19.07 ± 0.04	Mielec ^a^
BB9	5	5	2.5	5	5	10	20	>20	10	10	17.15 ± 0.25	nd
BB10	5	10	2.5	5	10	10	20	20	10	10	19.50 ± 0.33	Brusy
BB11	2.5	2.5	2.5	2.5	5	10	20	20	10	10	18.98 ± 0.18	Częstochowa ^a^
BB12	5	5	2.5	5	10	10	20	>20	10	10	18.64 ± 0.13	Miłogoszcz
BB13	5	5	2.5	2.5	10	10	20	20	10	10	19.66 ± 0.13	Malbork ^a^
BB14	5	5	2.5	2.5	10	10	20	20	10	10	18.54 ± 0.11	Suchorzew
BB15	5	10	5	5	5	10	20	20	10	10	18.20 ± 0.30	Miłogoszcz
BB16	2.5	5	5	5	5	10	20	20	10	10	18.49 ± 0.29	Miłogoszcz
BB17	10	10	5	10	5	10	20	20	10	10	19.04 ± 0.26	Majdan Starowiejski
BB18	5	5	5	5	5	10	20	20	10	10	18.60 ± 0.46	Warka
BB19	10	20	5	10	10	20	20	20	20	20	17.70 ± 0.38	Modzele

The underlined samples were bought from shops; other samples were provided by beekeepers. “a” indicates that the apiary was located in an area near the presented city (not exactly in the city/urban area).

**Table 2:** Antibacterial activity of selected ethanolic extracts of BP and BB against clinical isolates of *Staphylococcus aureus*. Isolates 1–6 are methicillin-sensitive and strains 7–9 exhibit the methicillin-resistant phenotype.

Product	BP9		BP15		BP20		BB6		BB11		BB14	
Strain No.	MIC [*v*/*w*] [%]	MBC [*v*/*w*] [%]	MIC [*v*/*w*] [%]	MBC [*v*/*w*] [%]	MIC [*v*/*w*] [%]	MBC [*v*/*w*] [%]	MIC [*v*/*w*] [%]	MBC [*v*/*w*] [%]	MIC [*v*/*w*] [%]	MBC [*v*/*w*] [%]	MIC [*v*/*w*] [%]	MBC [*v*/*w*] [%]
1	10	20	10	10	10	10	5	5	2.5	5	5	5
2	10	10	5	5	5	5	5	5	2.5	2.5	2.5	5
3	10	10	10	10	10	10	5	5	5	5	2.5	2.5
4	10	20	10	10	10	20	2.5	5	2.5	5	5	5
5	10	20	5	10	5	10	2.5	2.5	2.5	5	5	5
6	10	10	5	5	5	5	2.5	2.5	2.5	2.5	2.5	5
7	10	10	5	5	5	5	2.5	5	2.5	5	2.5	2.5
8	10	10	5	5	5	5	2.5	2.5	2.5	2.5	2.5	2.5
9	10	10	5	5	5	5	2.5	2.5	2.5	2.5	5	5

**Table 3:** MSSA and MRSA strains used in this work.

No.	Number/Phenotype	Ward/Material	Antibiogram ^1^
1	4471313/MSSA	Intensive care/Nasal swab	Resistant—Pen. Sensitive—Met., Clin., Ery.
2	4475564/MSSA	Internal/Nasal swab	Resistant—Pen. Clin. Ery. Sensitive—Met.
3	4466686/MSSA	Surgical/Sputum	Resistant—Pen. Clin. Ery. Sensitive—Met.
4	4467080/MSSA	Internal/Nasal swab	Resistant—Pen. Sensitive—Met. Clin., Ery.
5	4467076/MSSA	Laryngology/A swab from the ear	Resistant—Pen. Sensitive—Met. Clin., Ery.S
6	4468505/MSSA	Interna/Nasal swabl	Resistant—Pen. Clin. Ery. Sensitive—Met.
7	45300223/MRSA	Pediatrics/Blood	Resistant—Pen. Clin. Ery. Met.
8	9935169/MRSA	Dispensary/Wound	Resistant—Pen. Clin. Ery. Met.
9	9944662/MRSA	Dermatology/Nasal swab	Resistant—Pen. Clin. Ery. Met.

^1^—Identification of bacterial isolates and determination of antibiotic susceptibility analysis performed by Laboratory of Clinical Microbiology, University Centre for Laboratory Diagnostics, Medical University of Gdańsk Clinical Centre with Vitek2 Biomerieux system; Pen—penicillin, Met—methicillin, Clin—clindamycin, Ery—erythromycin, R—resistance, S—sensitive.

## Data Availability

The data presented in this study are available on request from the corresponding author.
